# Play, attention, and learning: How do play and timing shape the development of attention and influence classroom learning?

**DOI:** 10.1111/nyas.12154

**Published:** 2013-06-13

**Authors:** James H Hedges, Karen E Adolph, Dima Amso, Daphne Bavelier, Julie A Fiez, Leah Krubitzer, J Devin McAuley, Nora S Newcombe, Susan M Fitzpatrick, Jamshid Ghajar

**Affiliations:** 1 Brain Trauma FoundationNew York, New York; 2 Department of Psychology, New York UniversityNew York, New York; 3 Department of Cognitive, Linguistic, and Psychological Sciences, Brown UniversityProvidence, Rhode Island; 4 Rochester Center for Brain ImagingRochester, New York; 5 Department of Brain and Cognitive Sciences, University of RochesterRochester, New York; 6 Faculty of Psychology and Educational Sciences, University of GenevaGeneva, Switzerland; 7 Center for Neuroscience, University of PittsburghPittsburgh, Pennsylvania; 8 Department of Psychology, University of CaliforniaDavis, Davis, California; 9 Center for Neuroscience, University of CaliforniaDavis, Davis, California; 10 Department of Psychology, Michigan State UniversityEast Lansing, Michigan; 11 Department of Psychology, Temple UniversityPhiladelphia, Pennsylvania; 12 James S. McDonnell FoundationSt. Louis, Missouri; 13 Department of Neurological Surgery, Weill Cornell Medical CollegeNew York, New York

**Keywords:** play, attention, learning, education, anticipatory timing, synchronization, infant development, locomotion, perceptual–motor coordination, action video games, architecture, isotropic fractionator, head-mounted eye-tracking, cortex, evolution, spatial skill, puzzles, child ANT, transfer, STEM

## Abstract

The behavioral and neurobiological connections between play and the development of critical cognitive functions, such as attention, remain largely unknown. We do not yet know how these connections relate to the formation of specific abilities, such as spatial ability, and to learning in formal environments, such as in the classroom. Insights into these issues would be beneficial not only for understanding play, attention, and learning individually, but also for the development of more efficacious systems for learning and for the treatment of neurodevelopmental disorders. Different operational definitions of play can incorporate or exclude varying types of behavior, emphasize varying developmental time points, and motivate different research questions. Relevant questions to be explored in this area include, How do particular kinds of play relate to the development of particular kinds of abilities later in life? How does play vary across societies and species in the context of evolution? Does play facilitate a shift from reactive to predictive timing, and is its connection to timing unique or particularly significant? This report will outline important research steps that need to be taken in order to address these and other questions about play, human activity, and cognitive functions.

## Overview of the workshop

“Play, Attention, and Learning: How Do Play and Timing Shape the Development of Attention and Facilitate Classroom Learning?” was a one-day workshop convened by the New York Academy of Sciences and the Brain Trauma Foundation on June 15, 2012 in New York City. The workshop explored the idea that the design of classroom-based learning activities implicitly builds on many of the cognitive abilities children typically acquire through informal activities earlier in childhood, including physical play. Neural connections that facilitate synchronizing temporal and spatial expectancy with incoming sensory information may be formed through certain activities requiring children to clap, hop, or perform other rhythmic actions dependent on anticipation and timing.

Hypothesizing a connection between play and the development of important cognitive abilities expands the notion that interacting with the environment—particularly when engaged in activities that rely on anticipatory timing, cadence, or actions linked to subsequent actions—shapes the development of the attention network. Engaging in play could provide the developing brain with spatially and temporally predictive interactions with the outside world and thereby tune the developing network’s ability to select which information to attend to when, and which information to ignore.

The main goal of the workshop was to review the current state of scientific knowledge and to make recommendations for future research priorities. The role of timing in play and the development of attention has not been a traditional focus in developmental neuroscience research. With recent interest in the role of predictive timing in attentional focus, the natural progression is to ask how this capacity develops and whether it has an impact on subsequent learning ability.

The workshop was organized into two sessions. In the first session, a series of overview lectures provided the participants with an introduction to the current state of knowledge about the role of play in children’s cognitive development from the perspective of different disciplines and experimental approaches. The second session consisted of break-out discussion groups charged with reviewing existing knowledge on issues posed by the workshop organizers (detailed below), brainstorming and identifying areas of converging research, developing possibilities for future work, and considering how these activities may aid in the design of interventions for children with attention-related and learning disabilities.

## Introductory lectures

Jamshid Ghajar (The Brain Trauma Foundation) gave the first of two brief introductory talks preceding the overview lectures. He described the genesis of the workshop and discussed research conducted by the Brain Trauma Foundation in people diagnosed with concussion, with an interest in the connection between attention and predictive timing. In collaboration with Richard Ivry at the University of California, Berkeley, he tested the hypothesis that predictive timing is an essential element of attention in dynamic interactions.^1^ This led to the question of how predictive timing, and thereby attention, develops.

**Figure 1:**
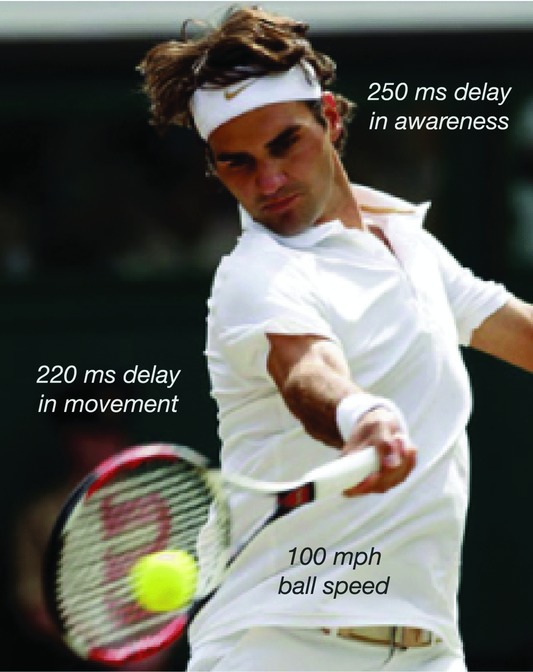
Tennis player, Roger Federer, is shown during a forehand stroke. Comparison of the latencies for sensory awareness and movement execution to the speed of the incoming ball highlights the necessity of predictive timing. The absence of accurate predictions for these varying delays would obviate making contact with the ball.

Synchronization with the outside world in dynamic interactions requires accurate predictive timing of the to-be-attended-to sensory information. It is important to be able to predict when relevant sensory events that are necessary for appropriate behavior will occur. Consider a tennis player who wants to hit a fast-moving ball but has varying latencies in sensory and motor processing (Fig. 1). To make contact with the ball, the player needs to predict when and where the ball is going to land, based, in part, on the motor actions of his opponent hitting the ball. Without the ability to predict when and where the ball will land, he would need to repeatedly swing his racquet to guess when the ball will arrive and would need to expend considerable resources to do so.

The predictive timing required to perform this racquet swing is analogous to that required in cognitive processing: to selectively attend, one needs to predict when the sensory information arrives, and which inputs are relevant to the situation, so that processing can occur efficiently and appropriate behavior can be generated. To make the connection between predictive timing and learning, consider a teacher and students in a classroom. To process the teacher’s words, the students need to predict the timing or the cadence of the teacher’s speech, allowing them to process the speech content just in time. Any deficiency in this kind of prediction could significantly impair their ability to listen and remember, which can manifest as a learning disability.

**Figure 2:**
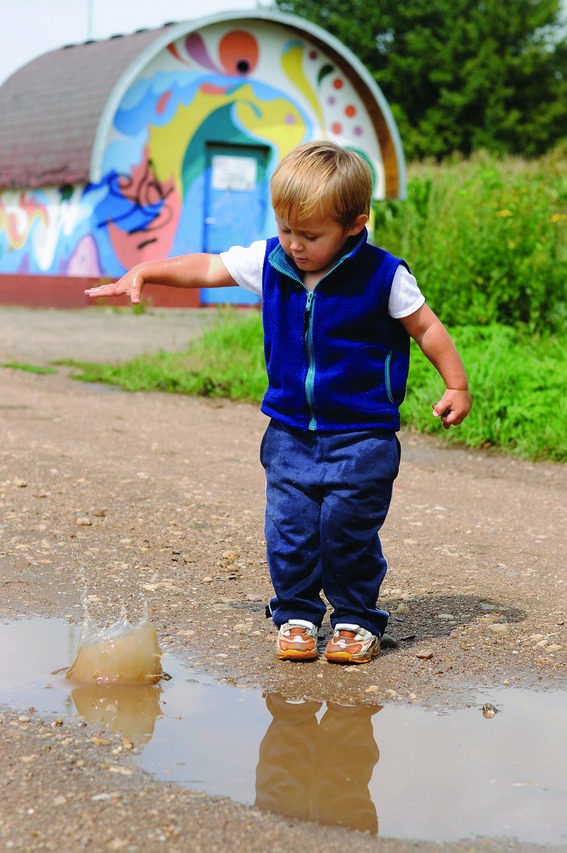
A boy is shown dropping a stone into a puddle. By repeating this predictable activity, he may develop stored representations of the properties of the external world from which accurate predictions of those properties can be formed.

Following these points, Ghajar considered what it means to play (i.e., how it can be defined). In his view, play may be a biological activity within which predictive timing develops. Play during early childhood coincides with cerebellar granule cell migration and synaptogenesis, and since the cerebellum has a known role in predictive timing, play may be the key to the development of this ability. Young children seem to seek out predictable interactions and then endlessly repeat them. By example, consider a young boy who repeatedly throws stones into a puddle (Fig. 2). He releases the stone, and after a certain period of time, there is a splash. He repeats this action until the expectancy of the splash matches the actual timing of the splash. This may be the result not only of reducing the variability in his motor process for throwing the stone, but also of forming better predictions of the stone’s spatial and temporal dynamics.

Following Ghajar, Susan Fitzpatrick (the James S. McDonnell Foundation) began her presentation with examples of the universality of certain aspects of play. Some childhood behaviors, such as playing patty-cake, have ancient roots and are common in many cultures around the world. These observations have led Fitzpatrick and others to speculate on whether some of the experiences common to play contribute to the abilities children call on for subsequent learning. It may be that some kinds of childhood activities should be considered a form of species-typical behavior necessary for the development of some of the brain networks required for successfully building the skills necessary for classroom learning. A first step in exploring these possibilities in future studies might be to determine the extent to which children engage in similar behaviors across cultures and whether play contributes to cognitive development.

Fitzpatrick is particularly intrigued with the many types of childhood play that are dependent upon timing. Consider the hand-clapping game “Mary Mack,” where there is an element of prediction: movements must be synchronized so that hands are in the right place at the right time. The game also builds on rhyming and song; children stand or sit opposite one another and clap hands in tune to the song. Timing is similarly important in “Double Dutch,” a game in which one or more players simultaneously jump over two long jump ropes that are turned in opposite directions. Predictive timing is key to jumping rope. In order to avoid getting hit by the rope, the jumper needs to know when it is going to arrive and how long it will take to execute his/her jump.

Fitzpatrick closed her talk by posing additional questions on how play could influence outcomes later in life. What might be the expected outcomes for children who do not engage in species-typical behaviors? When children are unable, for whatever reasons, to take part in the informal activities that might contribute to shaping the brain networks called upon in more formal learning situations, such as in a classroom, what kinds of activities could constitute this crucial form of play? What are the necessary elements? Could some children, struggling to make the transitions in early education, benefit from play-based interventions? Is it possible that the way skills are developed informally could be co-opted as a tool for building the skills required for success in more formal learning environments?

## Overview lectures

### Development of movement

Karen E. Adolph (New York University) initiated the expert overviews with a presentation entitled “Play and Human Development.” Adolph’s first point was that play occupies an immense part of an infant’s daily activity. Play—with objects, people, and features of the environment—generates a wealth of information. What are the opportunities for learning within play? How does the information that infants generate during play lead to learning? What kinds of information do infants take in and what are the properties of that information? Adolph and colleagues have worked to answer these questions within the context of locomotor, object, and social play.

The play of a typical toddler involves many different activities simultaneously: looking around, walking, holding objects, and interacting socially with others in the room. As Adolph showed in a series of video exemplars, head-mounted eye tracking combined with video tracking reveals a complex set of overlapping activities, with quick switches within and between activities, changes in speed, and continual starts and stops in locomotion.^2^–^3^ Eye gaze frequently switches among the targets of these simultaneous forms of varied activity (e.g., from a ball on the floor, to an upcoming obstacle, to a caregiver’s hands and feet); infants may pause to focus on the yellow ball or the doll in the scene.

Locomotor play is also extremely varied. Typically, infants’ movements cover an entire room, all the while engaged in different activities and looking at many different things.^4^ A graphic rendition of 10 minutes of spontaneous toddler activity reveals a twisting path that covers most of the locations in the room, with repeated loops through locations of interest. A raster plot of spontaneous walking from 60 infants confirms what we see in the video exemplars: locomotion is distributed in short bursts of activity, with longer periods of rest in between; each burst occurs in a different physical and social context.^4^ Infants’ spontaneous play can be described as “repetition without repetition,” an expression of the late Soviet neurophysiologist, Nikolai Bernstein.

Taken together, the above observations emphasize that any theory of learning has to work on multiple simultaneous streams of input. The relationships among the morphological characteristics of infants’ bodies, motor skills, incoming sensory information, and subsequent learning are multidirectional and dynamic. Methodological biases, such as an overreliance on looking measures, may obscure the complexity of these issues and may divert attention away from the extraordinary richness of babies’ behaviors.

Adolph argued that researchers should not rely on visual observation alone or assume that they know what features of the behavior are important. Rather, they should objectively collect and measure data at a number of levels, from eye tracking to walking. A case in point: using head-mounted eye tracking during free play to determine whether babies actually look, and how often, at their mothers. Although most researchers assume that infants look to their mothers’ faces, the data show that, in fact, infants look at their mothers only 54% of the time following mothers’ vocalizations; nearly half the time, they continue looking at whatever they are already looking at.^2^ When infants do look at their mothers, they rarely look at their faces and instead look at their bodies or hands. These findings were far more likely to be revealed through the new technology of head-mounted eye tracking than with visual observation alone.

Spontaneous locomotor activity increases sharply from experienced crawlers to novice walkers, and continues to increase in walkers from 12 and 19 months of age. Toddlers walk a lot; the average toddler takes 2368 steps/hour, which corresponds to roughly 14,000 steps/day.^4^ They can travel immense distances, about 701 m/hour, or the equivalent of 46 football fields/day. Object play is similarly varied, distributed, and immense. They touch many different objects, often all at once. Infants are in contact with objects for more than 30 minutes out of every hour, and there is a significant increase in object play between the ages of 11 and 13 months.^5^

The transition from crawling to walking affects what infants see. Thus, changes in motor skill lead to changes in play and corresponding changes in the input. While crawling, infants keep their heads down and look at the floor; people, toys, objects, and the facing walls are out of view. While walking, the whole room comes into view.^6^ The transition from crawling to walking also has a significant impact on object carrying.^7^ Walking infants carry objects much more than crawlers do (e.g., one walker carried objects 144 times within one hour). And although crawling infants also can carry objects (e.g., one crawler carried objects 20 times within one hour), they have a different way of doing it. Crawlers crawl while holding an object (e.g., under their arm or in their mouth) or while pushing it, or they bum-shuffle while holding an object in their hands.

Developmental changes in posture affect how infants share objects with their mothers and, more generally, how infants and mothers interact.^5^ Social interactions emerge from object carrying. Do infants carry objects to share them with their mothers? Yes they do. Crawlers share objects with their mothers, but primarily from a stationary position. They typically sit in one place holding up an object, and the mother has to come over to them to engage in the interaction. In contrast, walkers share objects while mobile. They pick up the object and carry it to the mother to engage in the interaction.

Differences in how objects are shared matter to mothers. Developmental changes in posture initiate a developmental cascade, such that mothers’ verbal responses to infants depend on how infants share.^8^ When infants sit in one place and try to share an object by holding it up, mothers typically respond by ignoring infants’ bids or affirming what they did, such as by saying “thank you;” less frequently, they name the object, such as by saying “oh, an orange ball;” sometimes they give action directives, such as by saying “put the block in first.” In contrast, when walkers carry objects to their mothers, the mothers respond with more action directives than any other type of response. It should be noted though that the difference in maternal responding is not merely due to an infant’s upright posture. In the few cases when crawlers carried objects to their mothers, the mothers responded primarily with action directives. In other words, postural development affects how infants share objects, and how they share objects, in turn, affects how mothers respond.

A similar cascade of developmental changes relates to 3-D form perception—knowing what the backside of an object is before you turn it around. Since objects are self-occluding, one needs to know what should be on the other side to perceive its 3-D form. A straightforward way to test 3-D form perception in infants is to habituate them to the frontal view of an object by rotating it a few degrees from side to side, and then test whether they look longer at an incomplete rather than complete version of the object when it is rotated 360°. If infants look longer at the incomplete version, it can be inferred that they perceived the full 3-D form during habituation.^9^

Applying this task, one finds that the ability to sit independently predicts 3-D form perception. Why might this be the case? Sitting frees infants’ hands, allowing them to rotate objects, transfer objects from hand to hand, and finger the edges of objects. These spontaneous play activities support the acquisition of a fuller representation of object form. In short, independent sitting gives infants more opportunities for visual–manual object exploration. Infants who cannot yet sit rotate objects in the same frequency with and without looking at them, whereas infants who can sit do much more exploration with looking. The ability to explore objects by looking and touching leads to more sophisticated object knowledge.^9^

Adolph closed by reviewing the central points of her talk: play is repetition without repetition; infant play is complex and involves switching between multiple parallel streams; developmental changes (e.g., in posture, body growth, or skill) shape how infants play, which thereby facilitates access to information. Before taking questions, Adolph answered one of her own: Why do babies play?—because they can, because it is fun, and because it is interesting.

Fitzpatrick asked what happens if infants cannot play. Adolph said that there is much redundancy to increase the likelihood that they will be able to, but that if they cannot, they will not generate as much information for themselves, and opportunities for learning will be curtailed. Leah Krubitzer (University of California, Davis) commented that play is the only thing that infants do and wondered whether adults continue to play as well. Adolph responded by pointing out that adults do continue to play, but that much of infants’ play is novel. Devin McAuley (Michigan State University) asked about 3-D form perception and whether it is important that babies are actually rotating the objects. Adolph answered that passive exposure to objects does not lead to the development of 3-D–object form perception; rotation is indeed critical.

### Development of attention

Bruce McCandliss (Vanderbilt University) gave the second overview lecture. He began by noting a delightful quote from Albert Einstein: “Understanding physics is child’s play when compared to understanding child’s play.” McCandliss pointed out that you could replace the second mention of child’s play in this quote with attention, since, in his view, attention is an incredibly complicated phenomenon. McCandliss presented a series of questions: How do attentional networks relate to play? How do we learn to attend? How does that change over the course of development?—which prompts the question of what matters in life experience. How would play impact these issues? Assuming play is important to the development of attention, how can we get more play into education?

To answer these questions, one could isolate an attention-related activity in someone’s behavioral repertoire (e.g., how well they do in attending to something) and connect that to changes in their brain networks—exactly the approach that some labs have taken. McCandliss pointed out that Michael Posner and Steven Petersen, then at Washington University in St. Louis, wrote an influential paper about attention that looked at how brain damage relates to particular deficits in attention.^10^ They proposed the idea that there might be different networks relating to different subsystems of attention (i.e., different syndromes relating to different patterns of damage). They identified three, somewhat distinct, subsystems of attention: alerting, defined as achieving a state of readiness; orienting, defined as the selection of information from sensory input; and executive attention, defined as detecting and resolving conflict between potential responses.^11^

McCandliss continued by noting that a further innovation by Posner and colleagues came by developing a paradigm, the Attention Network Task (ANT), that could be used to look at the principles of attention by pushing around a simple decision.^12^ The decision in the ANT is based on whether something is pointing one way or another, akin to judging the directions of pointed arrows in the arrow-based version of the Eriksen flanker task.^13^ By applying different cue and target conditions, the ANT allows for the quantification of orienting, alerting, and executive attention. Measures of each are based on how long it takes the brain to process different information (i.e., by comparing reaction times).

An alerting cue can decrease the amount of time it takes the brain to process information, in general, by about 50 milliseconds.^12^ An orienting cue can also accelerate information processing by facilitating the allocation of attention to a particular region of visual space, which lowers the time it takes to process a target in that region.^12^ A set of targets that conflict, by contrast, can slow things down, perhaps by demanding additional processing to resolve the conflict.^12^ One way to think of the latter is in terms of driving. If a global positioning system tells the driver to go one way and a passenger in the car recommends going another way, it can take additional time to process that kind of discordant information.

McCandliss pointed out that the ANT has been adapted for young children by replacing the standard arrows used for adults with oriented cartoon-like fish.^14^ Results from the child version of the ANT revealed a developmental progression for alerting and orienting attention between four and seven years of age.^14^ This progression parallels the development of the frontal system, which enables greater cognitive control. Conflicting information, however, still generates significant problems for children in this age group.^14^ It can result in a doubling in reaction times and a fivefold increase in errors. Related imaging work suggests that conflict is represented in the anterior cingulate cortex (ACC) and the dorsolateral prefrontal cortex (DLPFC),^15^ structures that are late to develop and, in cases of parental conflict, can cause greater challenges in adults. Recent work has also explored how event-related potentials (ERPs) relate to developmental changes in young children.^14^ These results suggest that children’s brains are more sensitive to conflict, and that this sensitivity is progressively diminished as they develop into adulthood.

McCandliss referred to work by Adele Diamond showing that children can deal better with conflict if they engage in structured activities, especially those augmented with contextual information on the fly.^16^–^17^ Being engaged in something often leads to anticipation of when it is going to happen. The neural correlates of such anticipation consist of overlapping brain networks that are activated during the cue period of attention tasks (i.e., between the presentation of the cue and the presentation of the target).^18^ Different regions are activated for *getting ready* and for *handling conflict*. Anticipating an action that might have a conflict associated with it evokes activity in the presupplementary motor area, which is thought to be involved in creating a copy of what you are going to see and what is going to happen. Such copies are continuously compared in a flow state where people can adapt their behavior. Regions of the basal ganglia that facilitate millisecond timing are also thought to be involved.

In addition, contingent negative variation, an ERP component, has been shown to relate to expectancy.^19^ It can be used to predict how quickly someone is going to react to something, which therefore suggests that it is capturing some aspect of attention. McCandliss suggested that measuring contingent negative variation could be one way of identifying the signature of a shift from more reactive to more predictive processing. He expressed the view that these aspects of the development of the temporal dynamics of attention are likely important and yet remain largely unexplored. There could be a shift in the ability to predict with play, where children become more predictive, but additional work is needed to test this possibility.

McCandliss pointed out that advanced go/no-go tasks, such as the AX version of the continuous performance tasks (AXCPTs), have been used as another window into children’s cognition.^20^ To correctly perform an AXCPT, a subject presses a key after a particular letter, such as X, which is presented after another particular letter, such as A. The subject presses a different key after the presentation of all other letters. The time between presentations of letters varies but is predictable. Performance on the AXCPT shows a developmental progression between five and nine years of age. Children who are proactively engaging in the world differ in their performance from those who are not. Nine-year-old children also show an expectancy wave—a prediction of when each letter is going to show up, whereas younger children do not. Other signs of predictive ability on this task, such as pupil dilation, show up around eight years of age.

McCandliss changed directions and noted that play can also have an element of cruelty. Cruelty has been seen in play in the virtual game Cyberball, which is based on an Atari arcade game of seven-man American football.^21^ In some experimental settings, children play the game simultaneously while they are in a magnetic resonance imaging (MRI) scanner. They chose different teammates, a process that can make some of them feel excluded. During the exclusion phase, there is activation of the ACC that is more similar to activity seen during emotional conflict than during typical attentional activity. Activity in the subgenual ACC has been shown to be related to depression in 13-year-old girls.^22^

McCandliss ended by emphasizing that despite the above-mentioned insights, there is a period of development wherein little is known about attentional function, roughly between two and five years of age.

Alison Gopnik (University of California, Berkeley) cautioned that an assumption in the executive attention literature is that if successful adults do something, it must be good for children to do the same thing. Gopnik added that for the child version of the flanker task, if the focus is on getting the task right, then an observer would reach the conclusion that children do not perform well. But they may be trying to do something else, such as inferring a generalization of how many fish go in the same direction, and that they may be performing quite well, but doing so at the expense of the more adult-like objective of the task. In other words, infants may have the capacity to do something much more broad in relation to exploring their environment (than we might first think). Issues of this kind can be thought of as a trade-off between control and other factors, such as generalization. With this in mind, new tasks could be developed to push decision dynamics in new directions. Games could be developed that lack instructions, such as discovering as many fish as possible.

### Learning to attend

Daphne Bavelier (University of Rochester and University of Geneva) gave the third overview lecture, entitled “Learning to Attend: Lessons from Action Videogames.” Bavelier’s interest is in learning and brain plasticity, which she explores in the context of action video games, and in particular, first- or third-person shooter games. Action video games may be thought of as quite mindless, consisting simply of avatars running around shooting the bad guy; but practicing them, in fact, imparts benefits on a wide range of cognitive tasks.^23^ Playing action video games can have beneficial effects that include transfer effects to basic aspects of attention and to many other skills. They can, for example, affect visual attention and the efficiency of switching between tasks.

Bavelier listed a number of important questions: How does playing action video games affect how well one sees, or how well one can perform cognitive processes like mental rotation? And why does such a wide array of skills appear to be modified by playing these games? Are transfer effects a consequence of a general improvement in attentional control or, instead, the result of independent changes in each and every function? Toward answering these questions, Bavelier’s working hypothesis is that playing action games improves top–down attentional control and, in doing so, hones the ability of players to differentiate signal from noise. As a result, gamers are able to carry out more accurate inferences in the service of decision making and thus show more skilled performance on a variety of different cognitive tasks.

Bavelier pointed out that her work follows the Bayesian brain approach in which the human nervous system has as its main task to infer the most likely way to react given the information perceived, the stated task goal, and previous knowledge.^24^ Loosely speaking, this view holds that the brain at all times predicts what the best decision should be given the present state of the world and past experiences. For example, while someone is speaking, a listener’s brain will be engaged in predicting what the speaker is going to say based on the speech stream heard up to a given point, as well as the context of the discussion. Individuals who can make more accurate predictions will zoom in on the most appropriate decision faster. Bavelier’s lab has shown that, indeed, action video game play trains players to make more accurate decisions.

To illustrate how research in her lab proceeds, Bavelier presented a concise example from her work on changing vision for the better. Contrast sensitivity is the ability to distinguish small shades of gray; it is a fundamental aspect of vision that can, for example, make the difference between crashing and not crashing into the car in front while driving in a thick fog. Measuring contrast sensitivity with small sine-wave gratings, termed *Gabor patches*, is a common approach in vision science. The standard task consists of two brief presentations of small Gabor patches; the subject indicates whether the patch was there in the first or second presentation.

Bavelier conducted a study in which contrast sensitivity was measured before and after undergraduate students played video games for 50 h over the course of 10 weeks.^25^ Critically, participants were randomly assigned to an action video or a control game; in both cases, the games were selected from commercially available options that are known to be quite enticing. A major difference in the two types of games is that the control game did not have the same level of dynamics, including fast pace, the need for visuomotor control and divided attention, as well as the meshing of many goals and subgoals at many different time scales. Bavelier confirmed that subjects who played action video games had higher contrast sensitivity than control trainees. She also alluded to related work by Ian Spence and Jay Pratt at the University of Toronto that employed a similar group design and that established that playing action games enhances mental rotation skills more than playing control games.

Transfer effects from playing action video games may be partly explained by changes in attentional control. Data from Bavelier and others show that playing them sharpens top–down attentional control, such as selective attention over space, time, and objects.^26^–^27^ Bavelier and colleagues have found that habitual action video game players are better at suppressing distracting information and that the better the suppression, the faster the reaction time. This effect has been noted not just in young adults but also in children who engage in action video game play, although the details associated with the effects of playing at varying ages have yet to be explored. Bavelier mentioned that studies of child action gamers show that although spatial attention is matured by seven years of age, playing shooter games can change its developmental time course. Of the three types of top–down attention tested in children, each had a different maturational curve, but all were changed by action game play. Bavelier noted that given how ubiquitous video game play is among children in our society, what is called an experiment of nature is unfolding in front of our eyes, with possible repercussions on society that are currently poorly understand.

One of the techniques that Bavelier’s lab uses to better understand the source of attentional enhancement after action game play is called *steady-state evoked potentials* and consists of presenting subjects with four different streams of visual information and measuring patterns of activation.^28^ By flickering the information at different rates, each stream induces a unique pattern of activation over the participant’s scalp that allows one to measure, and follow, the fate not only of an attended stream of information but also of unattended, potentially distracting information streams. Using a similar technique, Srinivasan and colleagues also concluded that action gamers excel at divided attention and actively suppress distraction.^28^ In contrast, role game players efficiently enhance attended information but show no other changes. This and many other studies suggest that action gamers are not simply trigger-happy. Rather, they are dealing with incoming information more efficiently, whether visual or auditory. The most recent research that Bavelier described explores the possibility that by enhancing top–down attentional control, action video game play also fosters the ability to learn more efficiently.^24^

A key challenge for future research will be to determine the particular elements of action video games that are necessary for these effects. Bavelier believes there is a need for much more research, as the impact of the technology currently used throughout society is quite unintuitive. A recent study at Stanford University shows that undergraduates who report multitasking between many different media have very poor attentional control when measured in the lab; this is the case, despite these individuals being convinced they excelled at the laboratory tasks they were just evaluated on!^29^ In contrast, based on what Bavelier reported, playing action video games enhances attentional control, despite prompting an initial impression of being a mindless activity.

Bavelier closed by pointing out that playing action video games may thus be viewed as a tool for leveraging brain plasticity in various patient populations, be it in amblyopia or in reversing the cognitive consequences of aging. Playing video games is already under consideration as a training device/task for specific kinds of work. For example, laparoscopic surgeons that play action video games are better at performing surgery than colleagues who do not play them; they are more accurate and make fewer errors than colleagues without video play but with more experience in the surgery itself.^30^ Pilots or other military personnel may similarly benefit from such video game play. The application of this research to educational goals is also being considered; primary school children could use video game play to develop core abilities, such as number sense, object manipulation, or basic physics. More accurate representations within these systems could translate into better school performance.

### Development of the neocortex

Krubitzer gave the fourth overview lecture in which she explored how cortical phenotypes develop across lifetimes, and how it changes within them.^31^ Krubitzer’s lab considers the common features of brain organization in many different species due to homology. She also demonstrates that similar types of modifications have been made to the neocortex in a variety of different mammals, suggesting that there are significant constraints on how evolution builds the neocortex. Throughout evolution, brains change in highly predictable ways, and Krubitzer’s lab works to determine the factors that lead to specific phenotypic characteristics. One approach to determining which factors specifically contribute to phenotypic variability is to induce the types of changes to the developing brain that are thought to be contributing to evolutionary changes, and then examine the resulting brain organization. These types of studies allow one to postulate how transitions in phenotype occur.

Of course both genes and the environment play significant roles in generating the changes observed in different mammalian brains. Other major factors that contribute to phenotypic differences across mammals are the morphological and sensory specializations of an animal and the species-specific behaviors associated with specialized body parts.^32^ This specialization leads to an enlargement of sensory domain allocation (the amount of cortex devoted to processing inputs from a particular sensory system) as well as cortical magnification (an enlargement of the representation of the specialized morphology within a cortical field). Consider the duck-billed platypus (*Ornithorhynchus anatinus*), a semiaquatic mammal endemic to eastern Australia, as a different, and more extreme, example of morphological/behavioral specialization. When it interacts with the world, it closes its eyes, ears, and nose. Thus, the only sensory inputs relaying information about the environment are coming from touch and electrosensory receptors on its bill, an extreme magnification of which exists within its primary sensory cortex. In fact, most of its neocortex in general is taken over by inputs from its bill, roughly 75%.^32^

These observations indicate that remarkable changes in the neocortex can be effected by altering peripheral morphology, sensory inputs, and the types of behavior associated with a given sensory receptor system. The effects of altering peripheral morphology and sensory-driven activity on the cortical phenotype have been assessed in the South American opossum.^33^ This approach consists of removing all of their visual input and examining the effect of this loss on the functional organization and connectivity of their neocortex. There is no change in the size of their cortical sheet. However, what would normally be primary visual cortex (identified architectonically with myelin stains) becomes very small, but does not disappear. This is similar to what is observed in blind mole rats, which have very small eyes, covered by skin. They use their visual system only for setting their biological clocks, not for navigating the environment. Thus, with the loss of visual inputs, sensory systems associated with the lost system contract but do not disappear.

This complete loss of visual inputs in opossums also causes dramatic changes in the functional organization of the neocortex; all of what would normally be visual cortex is taken over by the auditory and somatosensory systems, and neurons in this area respond to somatosensory and auditory stimulation. Further, this cortex that would normally develop into visual cortex now receives connections from subcortical and cortical areas associated with processing auditory and somatosensory inputs.

The question is whether it is possible to direct this cross-modal plasticity following early loss of vision. The Krubitzer laboratory is beginning to answer this question by allowing opossums to develop in an enriched tactile environment following complete loss of vision, to determine if cortical plasticity can be directed and enhanced based on the sensory environment in which the individual develops. Will neurons be tuned to the enhancing stimulus? Will connections be modified such that inputs from somatosensory structures come to dominate the reorganized visual cortex? Can tactile discrimination be enhanced? In addition to examining the functional and anatomical differences that emerge with enhancement following sensory loss, the cellular composition of the reorganized cortex will be examined using the isotropic fractionator method,^34^–^35^ which involves homogenization of tissue, leaving cellular nuclei intact. Differential staining of these nuclei with DAPI (labels all nuclei) and NeuN (labels neurons) allows one to quantify whether neuronal versus non-neuronal cell numbers have changed and answer the question: Will there be more neurons in the enhanced brain and/or a greater density of neurons?

The role of early sensory experience in shaping cortical organization has been underscored by comparisons between the laboratory rat and the same species of wild-caught rat, meaning one that lives in a laboratory cage and one that lives out in the natural world.^35^ Measurable differences have been found in the size of their auditory and primary somatosensory cortex and in the neuronal composition of the primary visual area. Laboratory rats have a larger auditory and somatosensory cortex compared to wild-caught rats (senses that were probably the least impacted by laboratory rearing). However, wild-caught rats have a greater density of neurons in their primary visual cortex.

These results have led the Krubitzer laboratory to examine the effects of natural differences in social rearing in voles.^36^ Parenting styles of voles can be naturally divided into parents that have a lot of tactile contact with their young (high contact parents) and those that have significantly less tactile contact with their young (low contact parents). This contact occurs around the perioral facial area of voles (i.e., around the mouth and nose). Voles reared by high contact parents have a greater amount of primary somatosensory cortex devoted to processing inputs from the perioral facial region than those reared by low contact parents. In addition, there are differences in cortical connections between offspring reared by high versus low contact parents.

Genes also contribute to the cortical phenotype and impact cortical sheet size, cortical field size, cortical connections, and peripheral morphology. In addition, cellular mechanisms involved in plasticity can be genetically mediated. More complicated environmental factors, such as social learning and culture, also have a large impact and represent complex patterns of interacting sensory stimuli that impinge on the developing brain, generate changes in cortical organization and connectivity, and ultimately influence subsequent behavior. In all, brains develop to match the sensory context in which they develop and generate appropriate, context-specific behaviors.

### Development of educational ability

Nora S. Newcombe (Temple University) gave the fifth and final overview lecture, entitled “Play and Educational Outcomes.” Newcombe discussed how early spatial learning is important in subsequent entry into STEM fields (i.e., science, technology, engineering, and mathematics) and how spatial abilities can be improved. Newcombe reviewed evidence from Wai, Lubinski, and Benbow^37^ on the abilities of high school students and predictions of their occupational interests and paths. Factoring out verbal and mathematical ability and other background factors, the likelihood that people pursue certain occupations has been shown to relate to their spatial ability. Students who subsequently enter STEM fields have higher spatial skills.

In collaboration with David Uttal, Newcombe^38^ performed a meta-analysis of training effects for five different classes of spatial skill. *Disembedding*, the ability to look for some specific pattern and pull it out, has the smallest effect size, and *spatial perception* (i.e., discern horizontal and vertical, with respect to gravity) has the largest. However, all of the effect sizes from their analysis are large enough to have practical importance. Their analysis leads to the following question: How high, in terms of spatial ability, do people have to be to major in STEM disciplines? Assuming the threshold for entering STEM disciplines remains the same, shifting the distribution with training may increase the proportion that can enter these disciplines. Additional work is needed to establish whether improvements in spatial ability actually have this effect when examined in rigorous randomized control studies.

One approach taken by Newcombe has been to compare men and women with high and low spatial ability who did or did not play *Tetris* over the course of a semester. Both women and men with high spatial ability improve more rapidly initially than they do later, although both continue to improve and do not generally reach a ceiling. Women with low ability initially improve more slowly than they do later. Comparing two groups that are equal on pretest assessments shows that the training group beats the other group on posttest assessments and on retests. Newcombe and colleagues have shown that these effects are durable.

Newcombe pointed out that according to the Wai *et al*.^37^ study, teachers in K–12 tend to have lower spatial ability while they are in high school. In other words, students who will become K–12 teachers tend to have lower spatial ability than their peers. This presents a problem: How do we get teachers to develop scientists? Newcombe described five activities that students can do that can help develop spatial abilities, including spatial books and poems, puzzle play, paper folding, block play, and shape sorters.

Spatial language is very important for nurturing spatial development. Istvan Banyai’s *Zoom* is an example of a book that can facilitate spatial learning by applying and exchanging spatial language. There are no words in the book and parents have to talk about what they are seeing in different scenes. The scenes are shown from different perspectives and unfold at different scales, so there are many opportunities to use spatial language to continuously explain what is going on.^39^

Jigsaw puzzle play can also foster spatial development, something that may differ by gender; particularly between two and four years of age, gender differences can be seen. The quality of puzzle play has been found to be higher in boys. For example, they can do more difficult puzzles, their parents are more engaged, and they use more spatial language. However, the quality of the puzzle has a greater impact on girls. Whereas boys perform higher regardless of puzzle quality, girls perform better if they are given harder puzzles. This hints at a complex interaction where what children bring to the interaction is important.^40^

Play with blocks is also important. Blocks can be arranged in different ways, which allows for comparisons between free play, play with prebuilt structures, and guided play. In free play, the blocks are just there to play with, whereas in guided play, there is a defined structure to work towards and an assembly diagram. Results suggest that if parents interact during play with blocks at all, they will use more spatial language, but they increase spatial language even more in the guided play condition. In other words, the mere presence of blocks increases the use of spatial language, and in correlational and longitudinal studies, spatial language is associated with higher spatial skill.^41^

Newcombe, Kathy Hirsh-Pasek (Temple University), and graduate student Justin Harris have developed an assessment of spatial folding that works for young children. Their results show that children do not usually perform above chance when they are younger than five years of age. However, engaging in this kind of activity more systematically might enhance spatial skill. Shape sorters are another important tool for improving and assessing spatial ability. Work with shape sorters shows that children often see only typical shapes; for example, they do not see a hole in a triangle. Typically, kids are shown only equilateral triangles; the result is they can think that all triangles are like these. A survey of the kinds of triangles that children are shown in math books up to the age of four years shows that they are all conventional triangles (i.e., equilateral triangles). In comparing guided play, didactic instruction, and enriched free play, Kelly Fisher has shown that guided play does the best in getting them to learn the correct definition of shapes.^42^–^43^ Catherine Tamis-LeMonda (New York University) commented that a combined effort by a child and a parent would allow a child to quickly reach the right answer, but that the child would not then show significant transfer.

Bavelier pointed out that people improve on different spatial skills with 10–20 hours of practice. She mentioned that there is other work on expert *Tetris* players suggesting that they excel at rotating *Tetris*-like shapes but not other shapes. *Tetris* experts may not engage any longer in the effortful process of mental rotation, but rather use lookup tables, having learned the mapping of shapes to board. This pattern of behavior suggests that transfer may be a U-shaped function. During the early phases of acquiring an effortful task, the need for attention and executive control may train such domain-general resources and benefit others, relatively different tasks allowing broad transfer. Yet when expertise develops, the complexities of the task are learned at a more procedural level, releasing effortful processing and enabling expert performance. The price to pay is that the knowledge is now much more specific, implying only limited transfer.

Newcombe replied to Bavelier by pointing out that there are a limited number of shapes in *Tetris*, and that there are different routes for performance improvement. One approach is to memorize all the shapes in all possible positions, which has a particular signature in ERP. This is the route that most people would take to becoming expert *Tetris* players, but there may still be some generalized transfer of mental rotation skills. The general idea is that the richness of the environment within a game is important and may determine the amount of transfer that can result from practice. Tamis-LeMonda pointed out that speed of learning and transfer can be in opposition.

Ghajar asked about time constraints in *Tetris* and in shooter games. He asked whether there are differential effects of play for engaging spatial ability within limited time constraints. Is there a separation between learning how to mentally rotate things and doing them in a very short time frame? Ghajar also asked about what is transferring. Do players anticipate better and show reductions in reaction time or do they do something else? Newcombe replied that the only related work that she is aware of related to these questions is on gender differences in mental rotation. The results of this work suggests that if timing pressure is eliminated, women are equal to men. But that turns out not to be true according to other studies, implying that there is more going on than just timing. Adolph added that infant control and anticipation are some the most important issues researchers are working on.

Bavelier pointed out that action video games have a rich temporal structure. Players have goals at many different time scales and are effective at taking all of them into consideration. Many action video games are built to allow the player a good scaffolding of knowledge and, at the same time, always keep the players in a world where they are challenged. In order to have a good game, it is important for players not to be able to develop a routine by which they can know what will happen next. Also, the video game industry has figured out how to build games with such a delicate balance, a feature that may make them not only maximally interesting, but also good learning tools. The notion of just-right challenges in the field of learning is not new, but it is very hard to quantify, and is clearly individual-dependent, making its implementation quite delicate.

Scott Eberle (Strong National Museum of Play) closed the discussion on time and anticipation by pointing out that there are other variables to consider, and that changing them may provide some insight into what it means to be playful. Chess players sometimes turn their back on the board, relying on their memory of piece position. Puzzle players sometimes turn a puzzle upside down so they cannot see the picture, relying on shape alone to fit pieces together. It may be that altering a game in a way that is novel and surprising is what it means to be playful. Playfulness, therefore, may be a way of expanding expertise to new abilities.

Gopnik connected things back to the definition of play. There seems to be two different dimensions that are often conflated: (1) play is something that children are engaged in independently or is something that involves others and has a didactic or pedagogic component; and (2) play is designed to accomplish a particular goal or is broad ranging and exploratory. These are very different dimensions. Results suggesting that kids do better in guided play than in free play may in part be a consequence of tapping into something where developing a specific skill is what is best for an individual; but one could arrive at a different result if doing something broader was beneficial and more specificity was limiting.

## Working group sessions

In the working group sessions, the participants were assigned to one of four groups, and three topical questions were provided to them by the workshop organizers. Following a discussion period, each group’s spokesperson provided a summary and opened discussion with all workshop participants. The posed questions and selected points from the discussions are provided below.

### Question 1: What current research evidence is there to connect play activities and the development of attention to the ability to learn in formal settings, like the classroom?

Dima Amso’s (Brown University) group added to earlier discussions about the definition of play and which aspects of attention might be important for classroom learning. Play is an active and emergent process of interaction with the outside world. It encompasses exploratory processes, which subsequently give rise to either serendipitous or intentional discovery of something that may be important. Additional bouts of exploration within play may follow in a cascade, as one comes into contact with the world and discovers additional things to work on. There is a balance between mastery and exploration, with novelty engaging attention. Motivation, reward, and pleasure are also likely to be involved, because they all foster play and may support sustained attention on one task. In other words, these elements may add drive when working out a problem. Variability and repetition may also be particularly important for fostering play that will develop attention.

Specific kinds of play may connect to specific kinds of attention. Pretend play may, for example, facilitate the ability to consider someone else’s perspective and to switch between different tasks, whereas puzzle play may facilitate sustained attention to a difficult assembly task. Similarly, different aspects of attention may relate to different activities in classroom learning. Sustained attention may, for example, connect to listening to the teacher throughout a lesson. Task switching that is facilitated by play would allow for working on one problem set and then another without a big drop in performance. Similarly, planning ability from play would allow for such things as picking up particular objects while in the process of assembling a model toy. A related observation is that planning becomes more abstract as children develop. They begin to use rules to plan their play, and this transition would seem to, at least in part, relate to the development of the frontal system.

Other evidence on the connection between play, attention, and learning comes from comparisons of curricula and outcomes of Montessori education with those of the Tools of the Mind curriculum. Montessori centers on self-construction by means of interaction with the environment and on the idea of an innate path of psychological development. Montessori programs allow children to make their own choices within structured environments. Montessori emphasizes keeping activities challenging. Once a task becomes easy, the child goes on to the next level. This is repeated until they master a skill, at which point they move on to something else. Although Montessori may undervalue social interaction, it facilitates the development of attention even into adolescence. Tools of The Mind centers on the development of self-regulation (i.e., executive function). Executive function can be defined as the ability to regulate social, emotional, and cognitive behaviors. Certain interventions with young children promote executive function, which in turn correlates with children’s achievement in literacy and mathematics. In comparison to Montessori, children have much more time for pretend play, which may be particularly important for developing executive function.

Bavelier’s group added another definition of play: activities that are fun, voluntary, and flexible. Such activities involve active engagement, the absence of extrinsic goals, an element of pretend, and are usually done in an environment where there are very few consequences. Bavelier’s group pointed out that there is very limited evidence connecting play to cognitive ability, including attention, if the above definition of play is applied. However, evidence suggests that children who engage in free play have better self-control, an observation that connects more to Montessori than to Tools of The Mind. The latter is more contained and takes place in a restricted environment. In light of this point, Bavelier’s group also pointed out that it would be helpful to know what would happen if children played in an environment designed mostly by themselves.

Bavelier’s group continued by discussing other observations that suggest that if children engage in an activity frequently during free play, they will get better at it. Play that involves a lot of language interactions will, for example, make children all the more ready for language tasks. Play that involves organizing objects by number or by manipulation into different groups will, for example, build a better sense of numbers and numeracy. A similar link among attention and executive control does not seem to have been developed, although related work on discovery-based learning suggests that it can be important to ensure that children understand the underlying conceptual framework of a problem rather than just knowing how to solve it. This approach, with an emphasis on concepts, takes a long time, which is consistent with a trade-off between knowledge acquired and time spent on a task. A final point is that intrinsic and extrinsic motivation could be important factors. There are cultures in which children are not allowed to play by themselves in that all aspects of play are directed.

Julie A. Fiez’s (University of Pittsburgh) group introduced a third definition of play, one that takes a more comparative species approach and which can be described in terms of specific criteria: activity that is not immediately functional; is pleasurable; occurs in a relaxed field; is repetitive, but not stereotyped; and is spontaneous in nature. Each of these criteria can be evaluated across different species and along a developmental trajectory within one species. This definition prompts a number of issues, including the kinds of situations or activities that increase the likelihood of play and whether they encourage the exploration of variability and the causal mechanisms for initiating play. Species that use learning as one of their core survival mechanisms tend to have more extended play over the course of their development. Play allows for the opportunity to learn to make predictions and to reason about variability and causality in the world. In addition, in linking certain types of play to certain types of improvements, play that involves a lot of social interaction will lead to improvements in social interaction, whereas play that includes a focus on causality will show transfer to other processes involving reasoning about causality.

Newcombe’s group pointed out that there are many different kinds of play (e.g., puzzle play, swinging on a swing, pretending to be a fireman) that may have effects on attention. In fact, each type of play would seem likely to have an impact on what follows in substantive ways. Yet, importantly, little is known about the transfer problem in most cases. For example, it is not known, even from correlational analyses, whether children who are more likely to become firemen as adults spend their playtime in activities such as bouncing around playing a drum. Also, a distinction should be made between extreme environments and normal variation.

Suzanne Gaskins (Northeastern Illinois University) pointed out that attention could be defined differently than it usually is within the cognitive neuroscience community. Instead of the three subtypes measured in the ANT, attention could be considered to be something one does to survive in the street of a poor neighborhood. This alternative notion of attention connects to well-known work by Walter Mischel, Yuichi Shoda, and Monica Rodriguez at Columbia University on delay of gratification. It may be that for those people who develop in a low socioeconomic status or a war zone, it would make sense for them to “take the cookie” (i.e., not to delay gratification). To the extent that an educational process emphasizes, or draws upon, certain abilities that may be practiced within play, play may confer benefits. Yet evidence for this on play in the classroom is not available. Questions to be explored include whether children learn better from play than from direct instruction, and how structured play relates to learning.

### Question 2: What is the role of timing (i.e., rhythm and cadence) in play activities, and how might this influence the development of attention and learning?

Amso’s group pointed out that rhythms that are in the world include repetition, something that may be important for learning. Repetition provides a lot of information that may obviate the need for sophisticated attention systems early in life. It may be significant that the repetition that is exploited in children’s play can span multiple modalities. For example, patty-cake, the game in which two players clap hands while singing an English nursery rhyme, involves synchrony across motor, auditory, tactile, and proprioceptive inputs. This multimodal source of repeated information may be particularly important for learning.

Input following a regular temporal pattern may allow children to build up a structure for processing incoming information. Pervasive and persistent temporal patterns allow for predictions, which in turn allow for the creation of error signals, a key element of learning. That which is external may become internalized, and may help make predictions about what is going on. This can be thought of in terms of cascades of “I know this is coming up and then it did.” Related work by Michael Goldstein and colleagues at Cornell University emphasizes the role that social interactions play in learning and timing. If a mom has headphones on and cannot hear what her baby is saying but still responds at the right times, the baby will still learn, thus emphasizing the significant role that timing and cadence play in the early environment. An open question is whether this sort of timing requires motor patterns.

Bavelier’s group agreed that timing is important in play and that it helps to structure it. For example, play allows for social interactions to become highly structured. As above, timing makes play more rewarding by facilitating the creation of scaffolding for performing cycles of prediction, error prediction, and error measurement. In other words, it allows for the collection of feedback. But timing is not the only thing that can be used for structuring play; spatial information plays a significant role as well. Bavelier’s group pointed out that related work suggests that the dopamine pathway is important for controlling reward-based learning and decision making. An interesting element from this work is that the dopamine pathway is important not just for getting rewarded for getting something right but for intrinsic reward as well. Predicting which reward is expected releases dopamine at the time of the prediction, not at the point of experiencing the actual rewarding event.

Although the link between timing in play and learning is, in some cases, clear in the opinion of Bavelier’s group, the link between timing in play and attention is not. Studies on training with an interactive metronome, for example, do not make a connection to attention. Similarly, although there is an important push in social interactions for synchronicity (e.g., in joint attention), it is not known whether such synchronicity actually leads to greater joint attention. To this point, there is some evidence that interactions that seek to draw attention together are more common in cultures where mothers pay less attention than in cultures where they pay more attention—in other words, in cultures where children are used to receiving attention. And although it is unclear how this translates to effects on different aspects of attention and learning, it suggests that context is going to be important and is paying off in the timing of the interaction.

Fiez’s group asked whether timing is particularly special for play or whether it is just a pervasive element for species living in an environment. Some consensus went toward the latter, but it was acknowledged that play that centers on timing and cadence does tend to be particularly attractive and may be especially engaging. Language play, for example, may be likely to involve play where timing is important, although this type of reasoning starts to become teleological, blurring what is causing what. A similar idea was discussed on the connection of play to learning and attention. Skills that children practice through play will lead to improvements in those skills, but that is not a special benefit only for timing. The basal ganglia system stands at the intersection of timing prediction and reward or pleasure signals. To the degree that this system supports a biological clock, individual differences in timing and in the ability to synchronize timing may be important in maintaining interindividual play interactions. From research in rats and playground behavior of children, it seems plausible that this could be an important mechanism for generating productive play experiences.

Newcombe’s group pointed out that timing is obviously important for play activities and the development of attention and learning; almost everything has a temporal component: neuronal activity changes in time; rhythms are known to help memory and guide the temporal allocation of attention; rhythmic movements begin at the fetal stage; and contingent timing helps attribute agency to a partner.^44^–^45^ McAuley added that understanding the role of timing and cadence in play, and the consequences for development, requires consideration of the range of rates (tempos) in which a child is able to perceive cadence and track events in time. Notably, we live in a particular temporal world where if successive events are too separated in time, they are perceived as isolated events; for adults, this temporal integration window is about 2–3 s, whereas for children, this window seems to be closer to 1 second.

Moreover, within this temporal integration window, children are generally tuned in to faster tempos than are adults, with preferred tempo slowing across the life span. The implication for play and attention here is that if a child has a narrower range of tempos within which she can connect two events in time and a faster preferred tempo, this places developmental constraints on how well a child will be able to track events in time. In this regard, rhythmic play activities may serve an important function because they have the potential to entrain attention to the time scale of the engaged play. Hierarchically structured play activities, including those involving music, have the added advantage that they may help bootstrap the development of attention to increasingly longer time spans.

Discussion of this question closed by connecting to a different question: Is there a reason to think that an activity such as rhythmic training (with music training as a specific type) could be helpful in developing attention or in learning? Could such training expand the trainee’s temporal window of integration (i.e., the time scale with which they connect and track events)? Work on these questions suggests that music training can slow preferred tempos,^46^ which is to say the speed at which information processing is optimal changes and becomes more matured. Related work by Nina Kraus and colleagues at Northwestern University suggests that musical training can impact learning; and work at the Temporal Dynamics of Learning Center at the University of California, San Diego suggests that training in Gamelan drumming can impact attentional ability.

### Question 3: How can we apply what we currently know about the relationships between play, attention, and learning to better design early interventions for children with attention and learning disabilities?

Amso’s group suggested that, assuming that there is sufficient evidence linking play to development, one approach to answering this question would be to look at different trajectories following different kinds or amounts of play. One advantage to using play as a means of improving particular skills is that it is fun and often more engaging than straightforward practice. Tamis-LeMonda added that even if research confirms the importance of play, bringing it to public policy is going to be incredibly difficult: convincing schools or therapeutic teams that they should switch from practice to play would be difficult and something the field would need to tackle.

Bavelier’s group described a different approach to this question, which is to consider a specific disorder (e.g., autism), and ask what is known about the effects of play interventions. Of relevance to this approach are experiments using the game *Second Life*, which requires identifying with avatars and interacting with others. A player identifies with an avatar that he creates from scratch and hence can be whatever he wants (e.g., a little boy can become a woman with long eyelashes). One idea proposed putting autistic people “on their own island” and recording their interactions (i.e., how much they are looking at each other); in fact, over a period of six months they become more social. Bavelier’s group added that other results suggest that autism is not a problem of attention and not related to play in any way, but instead, that it may involve a lack of maturation of the gamma-aminobutyric acid (GABA) receptor during critical developmental periods. As a consequence, the GABA receptor ceases to be excitatory, which may be associated with the development of autism. There are intriguing studies supporting this in which antidiuretics are given to autistic children; these drugs act on the GABA cascade and have been argued to confer significant improvements within a few days of taking them. Considering another disorder, dyslexic children suffer from phonological problems, but they also seem to have attention-related problems. Video games have been used to retrain attention of dyslexic children. The approach to this research is to first work on attention and the child’s phonological learning.

Bavelier’s group pointed out that another related area is the field of play therapy, which would benefit from carefully controlled assessments. Work in this area includes studies in which children who have difficulty focusing and do not enjoy reading are supported by a reader robot. The robot asks open-ended questions, which soothes the children and facilitates learning. Rigorous studies based on these kinds of approaches, however, have not yet been done. Fiez’s group added that more basic scientific research to address these issues would be informative, but also that such science should move toward building bridges between them. Specific funding that focuses on bridging these issues could be helpful, but there is some doubt as to whether, at this stage, a given funding review board would be able to differentiate among the issues to determine what is informative and whether it is likely to bear fruit. Fiez’s group commented that in discussing intervention, their impression was that a lot of intervention work is not conducted rigorously and may be prone to the Hawthorne effect in which observed effects are not a consequence of changes in a group’s behavior, but are actually related to the social situation of the experiment and the treatment the group receives.

Despite these limitations, there are good clues that could guide the choice of interventions that are likely to be effective. Focus should be placed on the zone of proximal development, the space between what learners can do with assistance and what they cannot do, and on play that taps into content or ability that is desired. Structured play activities may be particularly likely to yield benefits that generalize and are worth exploring further. Discussion on these issues also included consideration of a different approach where children are simply provided with more opportunities for play. For example, in the Finnish educational system, children have more time to play, an approach that has been applied in the corporate world and for adults at companies like Google, which provide less structured time in order to promote innovation and efficiency.

Newcombe’s group mentioned work by Cole Galloway at the University of Delaware on children diagnosed with cerebral palsy (CP) who benefit from using scooters to move around, which has wide-ranging cognitive effects. This highlights the importance of understanding the nature of any particular disability and its consequences on normal development. Discussion also included ADHD and the consensus that the specific causes of ADHD are not yet fully understood, and that it seems unlikely that undifferentiated play would provide much help. Children with ADHD may actually need more structured environments than those without ADHD. Another issue to consider is that cultures or institutions can have different definitions of attention or different ideas of how attention manifests, leading to differences in the number of children who are classified as atypical. A final point is that a large longitudinal study should provide greater insights into how play relates to learning and attention.

## Conclusions

The participants of this workshop considered many aspects of play, essentially different compositions of what play accomplishes. Play is an active and emergent process of engagement with the world, which encompasses exploratory processes. It is repetitive, but not stereotyped, and is spontaneous in nature. Along with play there are transitions in body size and sensorimotor development, each of which could facilitate a reciprocal developmental process during play.

It may also be that certain elements of play generalize, whereas others do not. Evidence was reviewed that shows that improvements in playing certain games generalize, probably by sharpening top–down attention. Longitudinal studies that look at trajectories following different kinds or amounts of play could provide crucial insights on many of these issues. A first question might be to explore what happens if children cannot play, or cannot play very much. Observing children in their natural environment and developing ways to quantify play behavior would be key to knowing how play varies across contexts.

Timing in play was considered in relation to the development of attention and the ability for subsequent learning. Dynamic temporal patterns, such as cadence in speech, could be important for learning and repetition. Timing is such a pervasive function that teasing out its contribution could be difficult. Certainly, performance improvement with practice involves transitioning from reactive to predictive timing, so different kinds of timing need to be considered.

There was some agreement that play could be used as an intervention in severe learning disorders. Playing certain immersive reality games has improved the social abilities of young people diagnosed with autism, who have reduced social play and impaired predictive timing abilities. There was also some agreement that unstructured play may not be beneficial for children diagnosed with ADHD. While some evidence exists for the utility of play intervention therapy in pathological conditions, there is little work describing the specific play dynamics that were necessary for therapeutic efficacy.

## Questions for future work

The workshop identified a number of important questions, which could be the focus of upcoming research:

The role of play in development

1. How is the development of attention and learning influenced by play, and by structured and unstructured play in particular?
2. How are the development of the cerebellum (e.g., granule cell migration and synaptogenesis) and the formation of cerebellar–cortical connections influenced by play?
3. How does play that is composed of particular combinations of activities relate to the development of a particular combination of abilities later in life?
4. What are appropriate metrics for assessing attention and learning in young children?
5. In what cases would children learn better from play than from direct instruction?

Variation in play

1. How does play vary across cultures?
2. Which elements of video games are important for improving attention and, if possible, generalizing to other cognitive functions?
3. What are the effects of playing video games on attention and social skills?
4. What is the role of play in an evolutionary context?

Play and timing

1. How does play facilitate a transition from reactive to predictive sensory processing?
2. How do neural networks that support anticipatory timing (i.e., those that underlie contingent negative variation) develop?
3. What are the differential contributions of spatial and temporal regularities in structuring play?
4. Does movement need to be connected to temporal elements in play in order to drive any effects on learning and on the formation of internal temporal representations?

Play as an intervention

1. How can the abilities that children typically develop through play be modified to facilitate improved learning in more formal environments?
2. Would children with attention or learning disorders benefit from play-based interventions?
3. Would rhythmic training facilitate improved attention and learning abilities?
